# Prevalence of *Cryptosporidium* spp. in Sheep and Goats in Jiangsu, China

**DOI:** 10.3390/vetsci11040144

**Published:** 2024-03-22

**Authors:** Cheng Cheng, Zhengrong Fan, Darong Cheng, Jianping Tao

**Affiliations:** 1 College of Veterinary Medicine, Yangzhou University, Yangzhou 225009, China; 13291399018@163.com (C.C.);; 2 Jiangsu Co-Innovation Center for Prevention and Control of Important Animal Infectious Diseases and Zoonoses, Yangzhou 225009, China

**Keywords:** sheep and goats, epidemiology, *Cryptosporidium* spp., risk factors, zoonosis

## Abstract

**Simple Summary:**

*Cryptosporidium* spp. is recognized as an opportunistic zoonotic parasite that infects humans, wild and domestic animals, and is also a major cause of diarrhea in humans and various animals. Knowledge of the distribution and genetic diversity of pathogens can lay the foundation for the prevention and control of diseases. This study investigated the prevalence of *Cryptosporidium* spp. infection and *Cryptosporidium* species in sheep and goats in Jiangsu province of China. The results may contribute to the prevention and control of *Cryptosporidium* infection in Jiangsu region.

**Abstract:**

Sheep and goats serve as crucial hosts for *Cryptosporidium* spp. and are primarily responsible for its transmission via the fecal–oral route. This can result in symptoms such as lamb weight loss, diarrhea, and even fatalities, leading to significant economic losses. Currently, there is a lack of scholarly research investigating the prevalence of *Cryptosporidium* spp. infection in sheep and goats specifically within Jiangsu province. This study collected fecal samples from sheep and goats, extracted their DNA, amplified target bands using nested PCR, sequenced the DNA, constructed a phylogenetic tree, and identified the genetic genotype. In total, 3 positive samples were identified out of 398 samples. Furthermore, the gene sequences of these samples exhibited significant homology with *C. xiaoi* in GenBank. The phylogenetic analysis revealed that the *Cryptosporidium* spp. parasites under investigation are phylogenetically related to *C. xiaoi.* Conducting epidemiological investigations and accurately identifying the species of *Cryptosporidium* spp. is of utmost importance not only for the mutton sheep farming industry in Jiangsu but also for the proactive safeguarding of human health.

## 1. Introduction

Cryptosporidiosis is a prevalent zoonotic protozoan disease resulting from *Cryptosporidium* spp. infection. *Cryptosporidium* spp. has the ability to infect more than 260 animal species, including mammals, birds, reptiles, and amphibians, causing a range of symptoms such as acute or chronic diarrhea and gastrointestinal inflammation, and in severe cases, it can lead to fatalities. *Cryptosporidium* spp. can induce diarrhea in individuals with immunosuppressive conditions, including children, and can lead to long-term, life-threatening diarrhea in AIDS patients [1]. Consequently, it is classified as a significant zoonotic parasite by the World Health Organization [2].

Presently, 47 species and over 120 genotypes have been reported in *Cryptosporidium* spp. [3,4,5,6], in which *C. parvum* is the most frequently reported pathogenic species of *Cryptosporidium* spp. [7]. The first recorded case of *Cryptosporidium* spp. infection in a goat was documented in Australia [8], and subsequent occurrences have been reported globally [9]. To date, a total of nine *Cryptosporidium* species/genotypes, namely *C. andersoni*, *C. baileyi*, *C. hominis*, *C. xiaoi*, *C. parvum*, *C. ubiquitum*, *C. bovis*-like genotype, *Cryptosporidium* cervine genotype, and *Cryptosporidium* rat genotype II, have been detected in goats [10]. *Cryptosporidium* infections in sheep have been reported globally from numerous countries [11]. Currently, 14 *Cryptosporidium* species/genotypes, namely *C. andersoni*, *C. baileyi*, *C. bovis*, *C. canis*, *C. fayeri*, *C. hominis*, *C. meleagridis*, *C. parvum*, *C. ryanae*, *C. scrofarum*, *C. xiaoi*, *C. ubiquitum*, *C. muris,* and sheep genotype I, have been detected in sheep [12]. Among these, the two major *Cryptosporidium* species responsible for zoonotic infections in humans are *C. parvum* and *C. ubiquitum* [13]. Additionally, a study reported that *C. xiaoi* was identified in two HIV/AIDS patients in Ethiopia [14], suggesting that it poses a potential threat to human health.

Sheep and goats are economic resources in China. In recent years, China has encouraged the use of a sheep and goat breeding model to convert this to scale. Sheep and goat production in Jiangsu province is primarily concentrated in cities such as Xuzhou, Nantong, Huaian, Lianyungang, and Suqian, forming two major sheep and goat industry belts: Xuzhou–Suqian–Huaian and coastal regions. The output of sheep and goats in three cities (Xuzhou, Nantong, and Yancheng) accounts for over 66% of the total output in Jiangsu province [15]. Nevertheless, the farming of sheep and goats is threatened by parasitic diseases such as nematodiasis, taeniasis, toxoplasmosis, and cryptosporidiosis, which not only slow down production but also pose health risks to farmers and consumers.

Currently, there is a scarcity of data and reports regarding *Cryptosporidium* spp. infection in sheep and goat farming in Jiangsu province. This study aims to determine the prevalence and species of *Cryptosporidium* spp. in sheep and goats in Jiangsu province. The results may contribute to the prevention and control of *Cryptosporidium* infection in Jiangsu region and also expand our understanding of the distribution and zoonotic potential of this diarrhea-related pathogen in sheep and goats.

## 2. Materials and Methods

### 2.1. Chemicals and Samples Collection

EasyPure^®^ Stool Genomic DNA Kit (TransGen Biotech Co., Ltd., Beijing, China), EasyPure^®^ Quick Gel Extraction Kit (TransGen), Premix Taq™ (TaKaRa Taq™ Version 2.0) (Takara Bio Inc., Kusatsu, Japan), and DL2000 DNA Marker (Takara).

**Figure 1:**
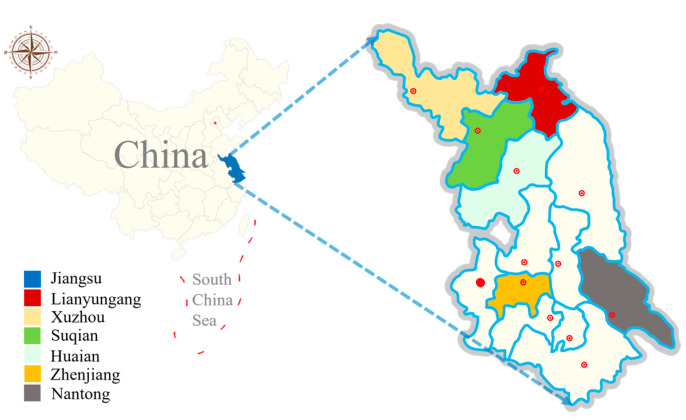
Distribution map of sampling districts of six cities in Jiangsu province of China.

From March 2021 to November 2021, a total of 398 fecal samples (274 from clinically healthy goat, 124 from clinically healthy sheep; sheep and goats fed in separate sheds) were collected from six large-scale farms across six cities of Jiangsu province. The climate in Jiangsu falls under a monsoonal climate, with the southern region characterized by a subtropical monsoon climate and the northern part by a temperate monsoon climate. Precipitation is abundant throughout the year, and the region experiences four distinct seasons (Figure 1). Out of 398 fecal samples, 206 samples were collected from animals aged 0 to 6 months, 124 were collected from animals aged 6 to 12 months, and 68 were collected from animals older than 12 months. Each sample, approximately 50 g, was placed in a clean sealed bag and labeled with information including the collection location, age, and a unique identifier. These samples were then transported to the laboratory and stored in a refrigerator at 4 °C for further analysis (within 48 h).

After the fecal sample was stirred evenly, half of each sample was placed in a clean, self-sealing bag and supplemented with a 2.5% potassium dichromate solution. Furthermore, 200 mg of each sample was preserved in 1.5 mL centrifuge tubes for subsequent fecal genomic DNA extraction, while the remaining samples were used for fecal parasite egg examination.

### 2.2. Extraction of DNA and PCR Analysis 

To perform DNA extraction, approximately 200 mg of fecal samples was placed into a beaker and mixed with a small quantity of sterile water. Subsequently, 250 μL of the resulting mixture was aspirated into a 2 mL centrifuge tube, and DNA extraction was conducted using the EasyPure^®^ Stool Genomic DNA Kit according to the manufacturer’s instructions.

For nested PCR amplification of the *Cryptosporidium* spp. SSU rRNA gene, primers were designed following the methodology reported by Xiao [16], and they were synthesized by Huada Gene (Table 1).

The first round of PCR amplification was conducted in a reaction mixture totaling 25.0 μL, comprising 12.5 μL of Premix Taq, 1.0 μL of each of the upstream and downstream primers (10 mmol/L), 1.0 μL of template DNA, and supplemented with double-distilled water to reach a final volume of 25.0 μL. The reaction conditions involved an initial denaturation step at 94 °C for 5 min, followed by 35 cycles of denaturation at 94 °C for 30 s, annealing at 56 °C for 30 s, extension at 72 °C for 1 min, and a final extension at 72 °C for 10 min. 

The second round of PCR amplification was conducted in a reaction mixture totaling 25.0 μL, containing 12.5 μL of Premix Taq, 1.0 μL each of the upstream and downstream primers (10 mmol/L), 1.0 μL of template DNA (diluted 10-fold from the first round), and supplemented with double-distilled water to reach a final volume of 25.0 μL. The reaction conditions included an initial denaturation at 94 °C for 5 min, followed by 35 cycles of denaturation at 94 °C for 30 s, annealing at 60 °C for 30 s, extension at 72 °C for 1 min, and a final extension at 72 °C for 10 min. 

Following the completion of the reaction, 10 μL of the second-round PCR product was loaded onto a 1% agarose gel prepared with TAE buffer. Electrophoresis was conducted at 120 V for approximately 30 min, and the gel was subsequently visualized and photographed using a UV gel imaging system (BIO-RAD, Hercules, CA, USA). The DL 2000 DNA Marker was employed as a reference. Positive bands were purified from the gel using the EasyPure^®^ Quick Gel Extraction Kit.

### 2.3. Phylogenetic Analysis and Statistical Analysis

The raw sequences obtained after sequencing were first aligned using Clustal X (1.83) software. The homology comparison of *Cryptosporidium* spp. SSU rRNA was conducted against the GenBank database using Blast. The relevant reference sequences were retrieved, and a phylogenetic tree for species identification was constructed using MEGA 7 software.

Statistical analysis was carried out using IBM SPSS Statistics 26 software, and chi-square tests were conducted to analyze potential factors influencing the infection rate of *Cryptosporidium* spp. in sheep and goats (*p* < 0.05 considered statistically significant). This helped identify the risk factors associated with *Cryptosporidium* spp. infection.

## 3. Result

### 3.1. PCR Amplification Results

**Figure 2:**
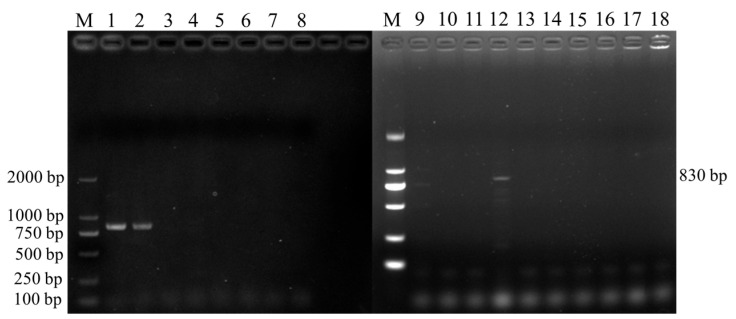
Results of *Cryptosporidium* spp. SSU rRNA gene PCR Amplification. M: DL 2000 Marker; 1~7, 9~17: fecal samples; 8, 18: negative samples.

In total, 3 positive samples were detected in 398 fecal samples collected in Jiangsu. The bands obtained through electrophoresis had an approximate size of 830 bp (Figure 2), consistent with the expected fragment size.

### 3.2. Sequencing Results

The PCR products were submitted for sequencing, yielding three *Cryptosporidium* spp. gene sequences. Sequence analysis using Blast indicated a homology of over 99% with *C. xiaoi* sequences from GenBank. 

### 3.3. Phylogenetic Tree Construction Results

**Figure 3:**
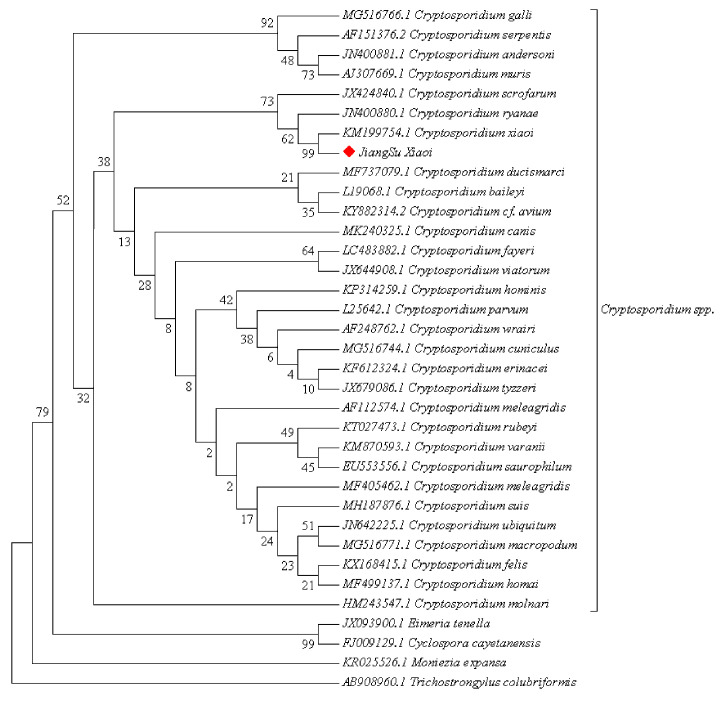
Phylogenetic tree of SSU rRNA gene of the *Cryptosporidium* spp.

A phylogenetic tree was constructed using MEGA 7, and phylogenetic analysis indicated that the *Cryptosporidium* spp. sequences discovered in this study cluster on the same evolutionary branch as *C. xiaoi* (Figure 3).

### 3.4. Infection Status of Cryptosporidium spp. in Sheep and Goats

The overall infection rate of *Cryptosporidium* spp. was 0.75% (3/398). The infection rate of *Cryptosporidium* spp. in goats was 1.09% (3/274), with no detection of *Cryptosporidium* spp. in sheep. All three positive samples were found in the same farm in Nantong city, resulting in an infection rate of 7.5% (3/40), whereas no *Cryptosporidium* spp. was detected in the Xuzhou, Zhenjiang, Suqian, Lianyungang, and Huaian areas (Table 2). *Cryptosporidium* spp. was only detected in lambs aged 0 to 6 months, while no *Cryptosporidium* spp. was found in sheep and goats aged 6 to 12 months and older than 12 months. Due to the low infection rate, there were no significant differences related with breed and age (*p* > 0.05) (Table 3).

## 4. Discussion

*Cryptosporidium* spp. is a globally distributed zoonotic protozoan. The ongoing development of sheep and goats farming in China, characterized by intensified farming, has elevated the risk of *Cryptosporidium* spp. infection in sheep and goats [17]. Presently, several regions in China have reported *Cryptosporidium* spp. infections in sheep and goats, with an overall infection rate ranging from 0.3% to 72%. Substantial variations in infection rates between goats and sheep are observed across different regions [18]. *Cryptosporidium* spp. oocysts are small in size, which poses challenges in species identification based on morphology. Additionally, traditional methods such as saturated salt flotation combined with acid-fast staining for microscopic examination yield a lower detection rate, increasing the likelihood of missed infections. In the study conducted by Wenchao Li et al. [19,20], microscopic examination was employed to analyze 83 sheep fecal samples and 781 goat fecal samples from Anhui province and neighboring regions. *Cryptosporidium* spp. was not detected in sheep, while the infection rate in goats was 0.26% (2/781). However, when using nested PCR, the infection rate in sheep increased to 5.8% (48/832), and in goats, it reached 8.7% (68/781). This highlights the significant improvement in *Cryptosporidium* spp. detection rates achieved through PCR technology, facilitating the effective assessment of zoonotic risks, species identification, and source tracing. Currently, there is limited research on cryptosporidiosis infection in sheep and goats in Jiangsu. In this research, we used nested PCR targeting the SSU rRNA gene of *Cryptosporidium* spp. to examine fecal samples from goats and sheep in various regions of Jiangsu province. The results revealed a *Cryptosporidium* spp. infection rate of 0.75% (3/398) in mutton sheep and goats in certain Jiangsu areas. This rate is similar to that found in goats from Henan (0.3%) [21], sheep from Henan (0.9%) [22], and sheep from Xinjiang (0.9%) [23]. However, it is lower than the infection rates reported in sheep from Ningxia (28.3%) [24], Sichuan (14.6%) [25], and Shandong (6.76%) [26]. While our survey indicates a relatively low *Cryptosporidium* spp. infection rate in sheep and goats in Jiangsu, it is important to consider that *Cryptosporidium* spp. oocysts are periodically excreted with feces, and infection rates may be affected by seasonal variations. Moreover, our sampling was conducted at a single time point, and the fecal samples were collected from large-scale farms with well-managed feeding practices. Therefore, it is possible that our investigation underestimated the *Cryptosporidium* spp. infection rate in sheep and goats in Jiangsu. 

Recent studies on *Cryptosporidium* spp. infection have indicated regional variations in *Cryptosporidium* spp. in sheep and goats in China. Research conducted in Guangdong, Shanghai, Hubei, and other regions has shown that the dominant *Cryptosporidium* spp. in infected goats is *C. xiaoi* [7], while in Henan and Chongqing, goats are primarily infected with *C. ubiquitum* [27]. In Henan and Sichuan regions, the dominant *Cryptosporidium* spp. in sheep is *C. parvum* [25,28], whereas in north China and northwest China, *C. xiaoi* prevails in sheep [14]. Despite the regional variation in *Cryptosporidium* spp. infecting sheep, *C. ubiquitum*, *C. xiaoi*, and *C. parvum* are the primary species responsible for sheep infections. In our survey conducted in Jiangsu, *C. xiaoi* was identified as the predominant species in infected goats. 

In a study led by Wenchao Li and colleagues, it was discovered that sheep fecal samples from Suzhou and Jiangsu contained not only *C. xiaoi* but also *C. ubiquitum*, specifically subtype 2. This subtype has the capacity to infect ruminant animals and has been associated with a significant number of human infections in countries like Canada and Spain, underscoring its crucial public health implications [21,27]. Future research efforts in Jiangsu province should include expanded molecular epidemiological investigations in farms to identify *Cryptosporidium* spp. and the subtypes involved in zoonotic transmission. This will serve as the basis for the prevention and control of *Cryptosporidium* infection in sheep and goats in Jiangsu province.

## 5. Conclusions

The prevalence of *Cryptosporidium* spp. in sheep and goats is relatively low in Jiangsu region, and the dominant species is *Cryptosporidium xiaoi*. In the future, it is necessary to conduct a more comprehensive investigation of *Cryptosporidium* spp. in a wider range of regions and farms with different feeding modes.

## Figures and Tables

**Table 1:** Primers for nested PCR amplification of *Cryptosporidium* spp.

Gene Locus	Primer	Sequence	Amplified Product
SSU rRNA	18S F1	5′-CCC TAA TCC TTC GAA ACA GGA-3′	1325 bp
	18S R1	5′-TTC TAG AGC TAA TAC ATG CG-3′	
	18S F2	5′-GGA AGG GTT GAT TTA GAT AAA G-3′	830 bp
	18S R2	5′-AAG GAG TAG GAA ACA ACC TCC A-3′	

**Table 2:** Prevalence of *Cryptosporidium* spp. in goats and mutton sheep.

Region	Sample Size	No. of Positive	% of Positive	Genotype (Count)
Suqian	126	0	0	-
Xuzhou	90	0	0	-
Lianyungang	82	0	0	-
Zhenjiang	44	0	0	-
Nantong	40	3	7.5	*C. xiaoi* (3)
Huai’an	14	0	0	-
Total	398	3	0.75	*C. xiaoi* (3)

**Table 3:** Infection of *Cryptosporidium* spp. in goats and sheep of different breeds and months.

Category		Total Tested	No. of Positive	% of Positive	*p*
Species	Sheep	124	0	0	Reference group
	Goats	274	3	1.09	>0.05
Age group	>12	68	0	0	Reference group
	6–12	124	0	0	>0.05
	0–6	206	3	1.46	>0.05
	Total	398	3	0.75	

## Data Availability

The raw data supporting the conclusions of this article will be made available by the authors, without undue reservation.
